# Oligomeric State and Holding Activity of Hsp60

**DOI:** 10.3390/ijms24097847

**Published:** 2023-04-25

**Authors:** Celeste Caruso Bavisotto, Alessia Provenzano, Rosa Passantino, Antonella Marino Gammazza, Francesco Cappello, Pier Luigi San Biagio, Donatella Bulone

**Affiliations:** 1 Department of Biomedicine, Neuroscience and Advanced Diagnostics (BIND), Institute of Anatomy and Histology, University of Palermo, 90127 Palermo, Italy; celeste.carusobavisotto@unipa.it (C.C.B.); antonella.marinogammazza@unipa.it (A.M.G.); francesco.cappello@unipa.it (F.C.); 2 Euro-Mediterranean Institute of Science and Technology (IEMEST), 90139 Palermo, Italy; 3 Istituto di Biofisica, Consiglio Nazionale delle Ricerche, 90146 Palermo, Italy; alessia.provenzano@cnr.it (A.P.); rosa.passantino@ibf.cnr.it (R.P.)

**Keywords:** Hsp60, monomer, oligomer, non-canonical function, amyloid aggregation

## Abstract

Similar to its bacterial homolog GroEL, Hsp60 in oligomeric conformation is known to work as a folding machine, with the assistance of co-chaperonin Hsp10 and ATP. However, recent results have evidenced that Hsp60 can stabilize aggregation-prone molecules in the absence of Hsp10 and ATP by a different, “holding-like” mechanism. Here, we investigated the relationship between the oligomeric conformation of Hsp60 and its ability to inhibit fibrillization of the Ab40 peptide. The monomeric or tetradecameric form of the protein was isolated, and its effect on beta-amyloid aggregation was separately tested. The structural stability of the two forms of Hsp60 was also investigated using differential scanning calorimetry (DSC), light scattering, and circular dichroism. The results showed that the protein in monomeric form is less stable, but more effective against amyloid fibrillization. This greater functionality is attributed to the disordered nature of the domains involved in subunit contacts.

## 1. Introduction

Hsp60 is a subgroup of the large family of heat-shock proteins. They are normally produced under physiological conditions and overexpressed under stress conditions. As essential components of the Protein Quality Control machinery [1], they control the proper folding of nascent proteins and prevent the aggregation of misfolded protein forms. They are also involved in cell signaling and protein transport across mitochondrial membranes.

The Hsp60 subgroup includes 60 kDa proteins that are generally assembled in a double-ring oligomeric structure. Together with co-proteins and ATP, they form macromolecular complexes, called chaperonins 60. These complexes act as true folding machines that recognize and interact with hydrophobic regions exposed by partially unfolded or misfolded proteins and use energy fueled by ATP hydrolysis to stabilize the folded conformations [2,3,4,5,6]. The best-known member of this group is bacterial GroEL, whose structure and functioning mechanism have been elucidated in great detail [3,4].

The homolog of GroEL in mammals is mitochondrial Hsp60, which is encoded by a nuclear gene, translated in the cytosol, and finally translocated into mitochondria after proteolytic cleavage of an N-terminal-target sequence [6]. The main function of mitochondrial Hsp60 is to support proper protein folding/refolding, in cooperation with Hsp10 and ATP. The mechanism of action was initially thought to be similar to that of GroEL. However, a series of experimental observations revealed some relevant differences at the molecular level. While GroEL is stably organized in two back-to-back heptameric rings, the formation of similar oligomeric structures in Hsp60 requires the presence of ATP and the co-chaperonin Hsp10 [7,8,9]. The reduced number of contacts between Hsp60 subunits [9] makes the tetradecameric structure less stable. The substitution of specific amino acids in the regions of inter-ring contacts leads to the lack of the negative inter-ring cooperativity [7,10] that distinguishes the efficiency of GroEL as a folding machine. Hsp60 is known to exert folding activity even in the single-heptamer conformation [7,10,11]. On the other hand, it is recognized that mitochondrial Hsp60 probably exists in equilibrium between monomeric, heptameric, and tetradecameric forms [8,12,13], and its structural dynamics underpin its unique functional versatility [9].

In mammalian cell lines, low levels of Hsp60 have been found outside the mitochondria, and it has been hypothesized that it may be involved in physiological activities other than folding assistance [14]. Under pathological conditions, a huge increase in Hsp60 has been observed in the cytosol, where it could exert pro-survival or lethal functions [13,15,16,17], depending on whether it is in monomeric or oligomeric conformation. An ambivalent role of Hsp60 was found in many cancers [18,19] and in several neurodegenerative diseases [1,5,20,21]. All these findings have generated a great interest in understanding the characteristic keys that regulate the multi-functionality of Hsp60 [22]. An interesting hypothesis is that the oligomerization state of Hsp60 could be related to its pathological or functional role outside the mitochondrion [12,13,23]. However, this information is missing in a large amount of literature data from in vivo and in vitro experimental studies.

In previous work [24], we observed that Hsp60 at a sub-micromolar concentration ratio strongly inhibits Aβ_40_ amyloid aggregation in the absence of ATP. We used a commercial recombinant Hsp60, whose arrangement in solution consisted of heptamers and tetradecamers in a wide range of protein concentrations [25]. The results suggested a selective action of Hsp60 against small, early aggregates of Aβ_40_, that would trigger the fibrillization process if left free in solution [24]. It was hypothesized that the mechanism might be sequestration of reactive amyloid species through some sort of holding action. The same type of mechanism was observed in evaluating the effect of a commercial recombinant Hsp60 on the fibrillization of Aβ_42_ in the absence of ATP [26]. The protective action of Hsp60 against the neurotoxicity of Aβ_42_ oligomers was demonstrated by in vitro and ex vivo experiments, and the physical interaction between Hsp60 and preformed oligomers of Aβ_42_ was thought to induce a conformational change of Aβ_42_ oligomers towards less toxic forms [27]. Additionally, in this case, a commercial recombinant was used.

Regarding the conformational state of Hsp60, much attention has been paid to heptameric and tetradecameric functionality in classical folding activity. However, it is becoming clear that issues of stability, structure, and the chaperone mechanism need to be investigated to elucidate the ambivalent role of Hsp60 in various diseases [28,29]. The ability of Hsp60 to bind hydrophobic patches is well-established [29], and both the flexibility and size of the exposed surface of the protein may be important for the interaction with hydrophobic species prone to aggregation.

Since the canonical folding mechanism, which requires ATP and a co-chaperone, does not seem to be involved in the protective effect of Hsp60 against amyloid fibrillization, the question arises as to what role the oligomeric structure plays here. Therefore, we wondered whether the same amount of protein in monomeric form could have a comparable effect. In this work, we investigated the relationship between the oligomeric conformation of Hsp60 and its ability to inhibit Aβ_40_ fibrillization in the absence of ATP. We produced a recombinant human Hsp60 and isolated the protein in monomeric or tetradecameric form to study and compare its effect on fibrillization of Aβ_40_. To rule out an effect due to different thermal stabilities, we also examined the thermal unfolding of the protein in the two conformational states.

To our knowledge, the present work is the first experimental study addressing the relationship between the chaperone mechanism and oligomeric conformation using biophysical techniques. The results should encourage researchers in the biomedical field to investigate the oligomeric state of Hsp60 and its impact on cancer or neurodegenerative diseases.

## 2. Results

### 2.1. Oligomerization State and Stabilizing Activity of Hsp60

**Figure 1:**
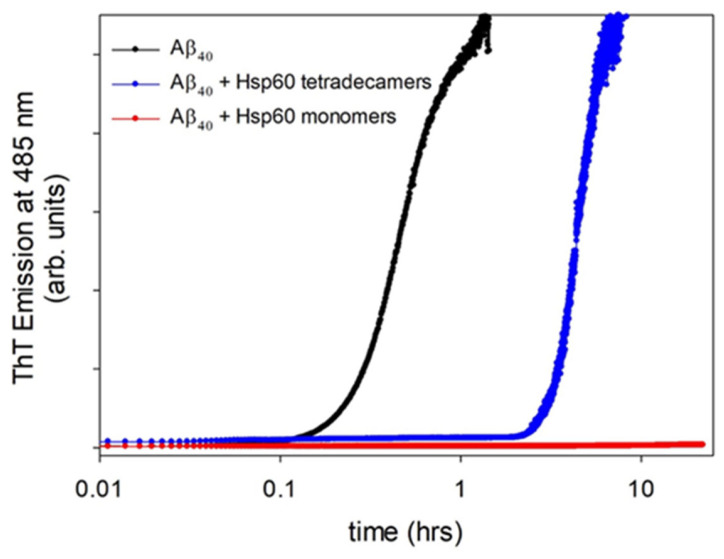
Time course of ThT fluorescence emission at 37 °C, for a sheared sample of 50 μM of Aβ_40_ peptide alone (black line) and with the addition of 2 μM of Hsp60 in oligomeric (blue line) or monomeric (red line) form. The wavelengths of emission and excitation were 485 and 450 nm, respectively. ThT concentration was 12 μM.

We used ThT fluorescence detection to follow the fibrillization kinetics of 50 μM of Aβ_40_ under destabilizing conditions (37 °C and stirring at 200 rpm), in the absence and the presence of a small amount of Hsp60 in monomeric or oligomeric form. The concentration of the protein in the two forms was expressed as the concentration of monomer units organized in oligomeric complexes or dispersed as single monomers in solution. Results are shown in Figure 1. In the absence of Hsp60, the typical, sigmoidal increase in ThT emission was observed. In the presence of tetradecameric Hsp60, the ThT signal increased similarly, but with a larger time delay. This indicates that Hsp60’s main effect is to inhibit the primary nucleation step [30]. Notably, when we added the same amount of protein in monomeric form, we did not observe any change, even after 24 h.

**Figure 2:**
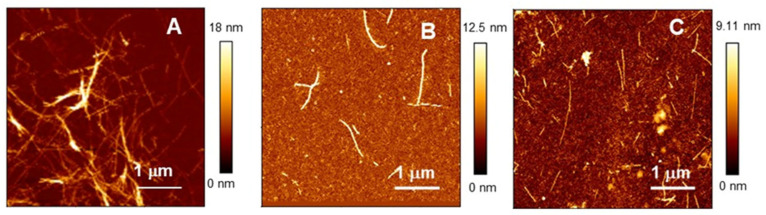
Tapping mode AFM images of samples of 50 μM of Aβ_40_ peptide alone (**A**) and with the addition of 2 μM of Hsp60 in oligomeric (**B**) or monomeric form (**C**). Note the different Z-scales. The images were taken at the end of the kinetics experiments of Figure 1.

The size and morphology of species contained in each sample at the end of kinetics experiments were analyzed by AFM imaging. Typical images of long fibril bundles were obtained in the case of Aβ_40_ alone (Figure 2A). Sparse isolate fibers with lower surface heights were detected in samples with tetradecameric Hsp60 (Figure 2B), whereas samples with monomeric Hsp60 displayed even thinner rare filaments, together with some globular denser objects (Figure 2C). When Hsp60, either in monomeric or oligomeric form, was added to already formed fibrils, no disaggregating effects were observed. As noted in our previous work [24], this indicates that the inhibiting mechanism acts before fibrils’ formation and might rely on recruitment of aggregation-prone Aβ_40_ molecules.

It should be noted that the final fluorescence intensity of ThT was comparable for samples without and with Hsp60 oligomers, although AFM images clearly showed fewer fibers in the sample with Hsp60. Since ThT is very specific for the cross-beta structure of amyloids, discrepancies between the data can be explained by differences in the structure of the aggregates.

### 2.2. Oligomerization State and Thermal Stability of Hsp60

The major efficacy of Hsp60 in monomeric form in binding aggregation-prone Aβ_40_ peptides might be conceivably related to the larger amount of surface available for interactions with aggregation-prone, partially unfolded, or misfolded molecules. On the other hand, the protein in the monomeric state might lose its thermal stability, as is well-known for other multimeric proteins, and then influence Aβ_40_ fibrillization in a non-native configuration. We applied DLS, DSC, and CD techniques to investigate this aspect.

**Figure 3:**
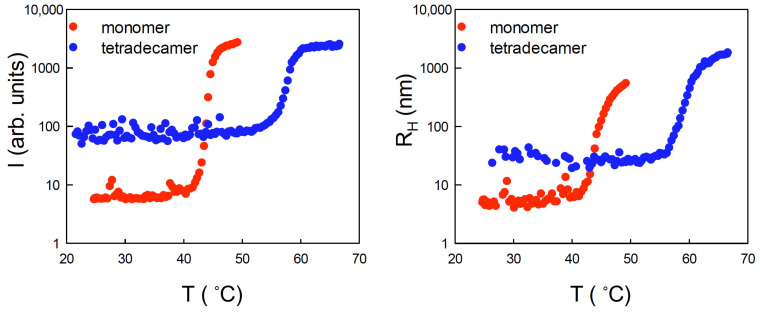
Static light-scattered intensity (**left panel**) and average hydrodynamic radius (**right panel**) vs. temperature for 15 μM of Hsp60 in oligomeric (blue symbol) and monomeric (red symbol) form.

Static and dynamic light scattering (SLS and DLS) measurements were performed on samples of 15 μM of Hsp60 in monomeric or tetradecameric form during a temperature scan. The intensity of scattered light and the mean average hydrodynamic radius of species in solution are shown in Figure 3 as a function of temperature. A sharp increase of the scattered intensity was observed at about 41 °C for the protein in monomeric form, and at about 54 °C for the protein in oligomeric form. The intensity increase correlated with a sudden growth of the hydrodynamic radius, from a few nanometers (4 nm and 24 nm for monomeric and oligomeric forms, respectively) up to several hundred. The accompanying growth of sample polydispersity and the appearance of multiple scattering, likely due to a rapid formation of large aggregates, made it impractical to continue the measurements. The irreversible formation of aggregates at high temperatures was confirmed by measurements on samples brought back at low temperatures.

**Figure 4:**
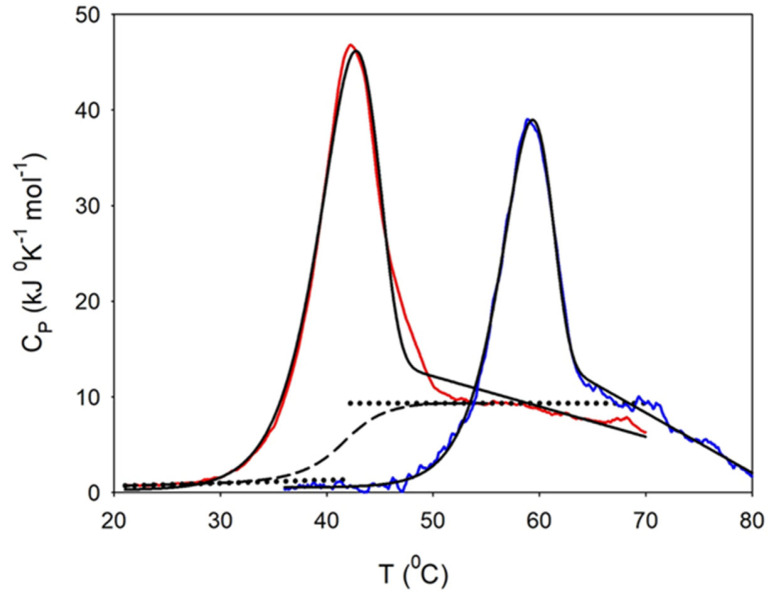
DSC thermograms of 15 μM of Hsp60 in oligomeric (blue line) or monomeric (red line) form. Calorimetric signals are given after subtraction of the instrumental baseline. The solid black lines show the best fit of the data to the simplest form of the Lumry–Eyring model. Additionally shown are linear extrapolations of the pre- and post-unfolding baseline (dotted lines) and the calculated chemical baseline (dashed line) for the signal of the protein in monomeric form. The scan rate was 30 °C/h. The thermal transition is not reversible for either form of the protein due to intermolecular aggregation. Notably, the monomeric fraction unfolds at a much lower temperature.

DSC thermograms for 15 μM of Hsp60 in the monomeric or tetradecameric form are shown in Figure 4. The protein in monomeric form unfolded with a T_m_ of 42.2 °C. The post-transition baseline was well-defined up to about 55 °C, and after that it slightly decreased, likely due to the formation of aggregates according to the DLS results. The contribution due to the difference between the heat capacity of native and unfolded proteins was calculated over the temperature interval 25–55 °C and subtracted from $C_{P}$ to obtain $C_{P}^{EX}$ . The integration of $\;C_{P}^{EX}$ over the same temperature interval yielded ΔH_cal_ = 318 kJ mol^−1^, which is 75% of ΔH_vH_ (425 kJ mol^−1^), calculated by Equation (2) (in Section 4.6). Large deviations of the ratio ΔH_cal_/ΔH_vH_ from unity evidence the inadequacy of the two-state model to describe the unfolding process [31]. In our case, a ratio slightly less than one could indicate the occurrence of partial aggregation according to the results from the light scattering measurements. Signals of aggregation were more visible in the DSC trace for proteins in tetradecameric form. The unfolding occurred with a T_m_ of 58.8 °C, but the peak was appreciably made asymmetric by the superposition of the well-visible drop in the post-transition baseline. This indicates that the aggregation process was quite fast in the unfolding temperature range [32].

Data analysis in terms of the simplest version of the two-state irreversible model (Equation (4) of Section 4.6) allowed deriving the unfolding enthalpy and the activation energy for the final step of the transition, from the unfolded to the irreversibly aggregated state. For comparison, the same analysis was applied to data for proteins in monomeric form, even if the fit quality was not as good. Fitting parameters are summarized in Table 1. No peak signal was observed in the downward temperature scan for the protein in either monomeric or tetradecameric form, thus indicating aggregation upon unfolding.

A larger thermal stability of the tetradecameric form was observed by Shao et al. [33] through differential scanning fluorimetry. The authors determined a large difference in T_m_ values (40.7 and 58.2 °C for monomer and oligomer, respectively), in good agreement with our T_m_ values. In comparison to GroEL [34], the thermal stability of Hsp60 was lower, likely due to the weakness of the inter-ring contacts.

**Figure 5:**
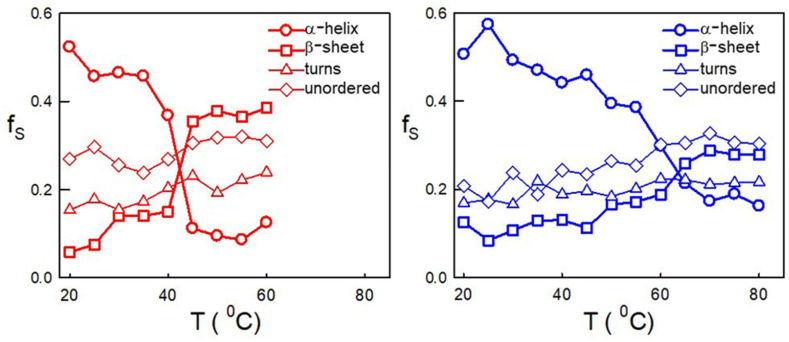
Fractions of secondary structure components vs. temperature for 15 μM of Hsp60 in monomeric (**left panel**) or oligomeric (**right panel**) form. Results in the figure were obtained by CDPro analysis of circular dichroism spectra at varying temperatures (see Figure S1).

Since the presence of monomeric Hsp60 in the cytosol has only recently become known, there is no information on possible differences in the secondary structure of the monomeric and oligomeric forms. In fact, one would expect some changes in the interfaces between the subunits [9]. To investigate this aspect, we collected far-UV CD spectra with increasing temperature for the protein in monomeric or oligomeric form (Figure S1). The signal at a 225 nm wavelength was also followed during the temperature scan. CDPro software [35] was used to determine the percentage contribution of alpha-helix, beta-sheet, turns, and disordered elements to any CD spectrum. As shown in Figure 5, the percent contribution of alpha-helix, beta-sheet, and the unordered structure was similar in both forms, whereas the percent contribution of the disordered structure was slightly higher in the monomeric form. In both protein forms, the percent contribution of alpha-helix decreased with the increasing temperature, while the percent of beta-sheet content increased. However, in the monomeric form, the change was steeper, and occurred at a lower temperature, with a lower final alpha-helix content and a higher final beta-sheet content.

**Figure 6:**
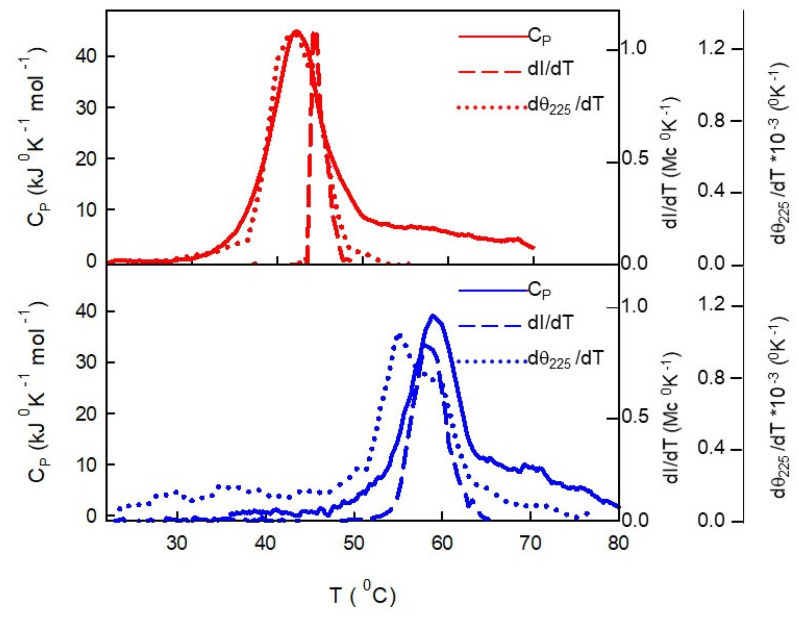
Comparison of the temperature dependence of DSC, LS, and CD results for 15 μM of Hsp60 in monomeric (red line) or oligomeric (blue line) form. For data consistency, the temperature derivative of molecular ellipticity at 225 nm and the scattered light intensity were plotted with the DSC trace.

The three sets of results from DSC, LS, and CD measurements are plotted together in Figure 6 to look inside the probable sequence of events. Note that the derivative of both light-scattered intensity and the molecular ellipticity CD at a 225 nm wavelength are plotted together with DSC traces to assure the consistency of the data. For the protein in monomeric form, the DSC signal and the derivative of the CD signal almost coincided, thus indicating that conformational transition and unfolding are simultaneous events. When the fraction of unfolded molecules was enough large (well beyond 50%), the scattered intensity began to grow, reflecting the formation of aggregates. A different scenario was observed for the protein in oligomeric form. In this case, the conformational transition preceded the unfolding signal, thus suggesting that conformational changes were still constrained in the oligomeric structures. When the oligomers finally melted, a concomitant formation of aggregates was observed, as reflected by the growth of the scattered light intensity.

## 3. Discussion

The different families of heat-shock proteins exert their protective action against misfolding and aggregation of biomolecules through specific mechanisms, generally related to their molecular weight [5]. However, members of each family are also capable of performing tasks other than their specific ones. For instance, small heat-shock proteins (sHsps) are known to exert a passive, ATP-independent “holding” activity, sequestering proteins in unfolded or misfolded form, and preventing their aggregation while awaiting the intervention of a folding chaperone. However, they are characterized by exceptional structural plasticity, which allows them to employ different mechanisms to control the binding/release of different client molecules [36]. In particular, an active conformational change from large oligomers to smaller units is associated with a dynamic low-affinity interaction for amyloid proteins [37].

An unusual activity that does not require ATP energy input is exerted by other ATP-dependent Hsps against amyloid proteins. Hsp70, known for its folding activity, prevents alpha-synuclein fibrillization in an ATP-independent manner by interacting with alpha-synuclein monomers through a binding site other than the canonical binding site for folding [38]. Hsp70 is also capable of inhibiting beta fibrillization at sub-stoichiometric concentrations without the aid of ATP and the co-chaperon [39] by recruiting small, early oligomers. Hsp90, known for its folding activity, binds to the fibril core region of tau protein, inducing the formation of small oligomers and inhibiting fiber formation [40,41]. Both Hsp90 and Hsp60 are able to exert non canonical, ATP- independent holding activity on proteins in misfolded form by forming multiple, weak, and nonspecific bonds. The target proteins retain their misfolded form but are protected from aggregation [42]. It has been shown that Hsp104, in addition to disassembling protein aggregates fueled by ATP hydrolysis, exerts ATP-independent holding activity on soluble monomers of amylogenic proteins [43].

Indeed, each member of the large family of molecular chaperons can employ different mechanisms to interact appropriately with different client proteins [44]. It is noteworthy that the ability to convert specific agents into appropriate defenders to cope with different events of the cellular life is a more efficient strategy than the production of specific chaperons. The multitasking ability, or moonlighting activity [45], is a property shared by proteins or molecules with disordered regions [46] that has attracted great interest in the search for new therapeutic strategies [45].

Hsp60 is known to function as a folding machine [2] with the assistance of co-chaperonin Hsp10 and ATP, in close analogy to the functioning of its bacterial homologue GroEL. Although the oligomeric (tetradecamer or heptamer) structure of Hsp60 is essential for folding activity, it is less stable than that of GroEL, and indeed, monomeric, heptameric, and tetradecameric forms can coexist in an equilibrium regulated by ATP and protein concentration [7,8]. The lower stability of the oligomeric structure of Hsp60 compared to GroEL is due to a smaller interface between subunits with a smaller number of intermolecular contacts [9,10]. Most of the contacts between subunits occur in the equatorial domain, where several amino acid sequences, unique to human Hsp60, make weak sidechain contacts upon ATP binding [9]. Interestingly, these specific sequences in the equatorial domain are even responsible for moonlighting functions [47].

Considering that the oligomerization of Hsp60 occurs only in the presence of ATP and nature never does anything by chance, it had been questioned if the protein in its dissociated form may have a physiological role [7,12,13,23]. The interest for what could be the role of a minor presence of Hp60 in the cytosol or other extracellular locations, and if the protein was in monomeric or multimeric state [18,23,48], was fueled by the findings of Chandra and co-authors, who observed that Hsp60 may exert pro-death or pro-vita effects in several apoptotic systems, depending on its localization and oligomeric configuration [13].

In this work, we investigated the mechanism exerted by Hsp60 against the amyloid fibrillization in relation to its oligomeric conformation, and we found that the protein in monomeric form was less stable but more effective in exerting a holding action, which is a task different from that commonly ascribed to Hsp60 in oligomeric form. A similar stabilizing effect at sub-stoichiometric concentrations was observed for Hsp90 on Aβ_40_ [49]. Interestingly, the authors found that the dimeric form was more active than the tetrameric form. This result was interpreted to mean that Hsp90 probably solubilizes Aβ_40_ monomers through weak interactions, the number of which is greater for the dimeric form, which offers a larger hydrophobic surface area [49].

In studying the effect of Hsp60 on the fibrillization of amyloid proteins, great attention has been reserved to the apical domain, which is the canonical region involved in the capturing of aggregation-prone proteins. Yamamoto et al. [50] had shown that a mutant of Hsp60, in which the apical domain is fixed in open conformation, was more effective than wildtype Hsp60 in inhibiting the fibrillization of a-synuclein. Although the mutant and wildtype forms have similar tetradecameric structures, the larger hydrophobic surface area of the open apical domain favors a stronger interaction with a-synuclein monomers, as confirmed by fluorescence assays with 8-anilino-1-naphthalenesulfonic acid (ANS) [50].

A major functionality combined with a less ordered and stable structure is a common feature of proteins having intrinsically disordered regions (IDRs) [51,52], whose plasticity enables a rapid response to external environmental changes [53]. Regions of structural disorder allow the molecule to interact with different partner molecules and perform moonlighting activity [45,54,55]. Several contacts between the equatorial domain of Hsp60 subunits are disordered regions. Conceivably, their exposure to the solvent confers monomeric Hsp60 the ability to interact with amyloid molecules to a greater extent than that due to the increase of the exposed protein surface. Further work is needed to elucidate the molecular mechanism by which Hsp60 inhibits Aβ_40_ fibrillization. Aβ_40_, similar to other amyloidogenic proteins, is an intrinsically disordered protein because it lacks the well-folded structure with minimum free energy that corresponds to the native state of a globular protein [56]. Rather, it can adopt multiple conformations with comparable, relatively low free energy [57]. Through a transient interaction, Hsp60 could select and stabilize specific conformations of Aβ_40_ that are resistant to aggregation and better-suited for binding or induce a conformational change that optimizes the interaction between the two molecules. These two mechanisms, termed conformational selection and induced fit, respectively, have been primarily hypothesized to describe how disordered proteins can undergo a major, irreversible conformational change after a transient interaction with other molecules [53]. However, the generalization of a precise model of action in multifunctional chaperons has been discouraged because the great versatility likely implies an easily modifiable, client-dependent specific mechanism [44].

Not much information on the possible relation between the oligomerization state and the biological function of Hsp60 or other chaperonins of the same family can easily be found in the literature, albeit there is continuous growth. To the best of our knowledge, the present work is the first in vitro experimental study on this topic. Studies on this relationship could provide insights into the controversial role of Hsp60 in cancers and neurodegenerative diseases [18,19,20]. We believe that our results can help raise awareness among medical and biomedical researchers, as knowledge of the molecular anatomy of the mitochondrial chaperonin Hsp60 is crucial for a better understanding of its physiological and pathophysiological roles in health and disease.

## 4. Materials and Methods

### 4.1. Plasmid DNA Construct

The gene encoding the wildtype human Hsp60 without the mitochondrial-targeting sequence (HSPD1, GenBank, Accession number NM_002156) was cloned into the pET-15b expression vector (Eurofins, Ebersberg, Germany). The HSPD1 sequence was inserted between the sequences for the restriction enzymes BamHI at the 3′ end and NdeI at the 5′ end. The resulting plasmid, pET-15b-Entry-HSPD1, encodes the recombinant Hsp60 as an N-terminal hexa-histidine tag protein.

### 4.2. Expression and Purification of Recombinant Human Hsp60

*Escherichia coli* BL21-Gold (DE3) cells (Agilent Technologies, Santa Clara, CA, USA) were transformed with pET-15b-Entry-HSPD1 and grown in Luria–Bertani (LB) broth soil plates (Sigma Aldrich Merck Darmstadt, Germany) containing agar (Sigma-Aldrich–Merck), with 100 μg mL^−1^ of ampicillin (Sigma), at 37 °C for 16 h. Then, 20 mL of LB broth containing 0.5% glucose (Sigma-Aldrich–Merck) and 100 µg mL^−1^ of ampicillin was used to prepare a pre-inoculum of a chosen colony. The pre-inoculum was allowed to grow overnight at 37 °C and 230 rpm, before being added to 1 L of LB broth containing 0.5% glucose and 100 µg mL^−1^ of ampicillin. When the optical density at 600 nm of the culture reached 0.6–0.8, expression was induced by the addition of 1 mM of isopropyl-β-D-thiogalactopyranoside (Sigma Aldrich Merck). After incubation at 37 °C and 230 rpm for 3 h, the cells were harvested by centrifugation at 2800× *g* for 10 min. The cell pellet was re-suspended in pre-chilled lysis buffer (20 mM sodium phosphate buffer, pH 7.4, 0.5 mM NaCl, 10 mM imidazole, 1 mM dithiothreitol, 5 mM MgCl_2_, 10 µg mL^−1^ DNAse, cOmplete EDTA-free protease inhibitor-Roche mixture, and 0.4 mg/mL of hen egg white lysozyme). The cells were disrupted on ice by an ultrasonic homogenizer (Bandelin HD 2070) and incubated at 4 °C for 30 min. The lysate was centrifuged at 20,000× *g*, 4 °C, for 30 min to remove cell debris and suspended particles. The supernatant was filtered through 0.45 μm filters (Sartorius Stedim Biotech, Gottinga, Germany) and loaded onto a 5 mL HisTrap FF column prepacked with Ni-Sepharose (GE Healthcare, CA, USA), equilibrated in the same buffer as the protein sample. The His-tagged protein was eluted on a FPLC system (ÄKTA pureTM 25 M, GE Healthcare) with a linear gradient from 10 to 500 mM of imidazole in 10 CV at room temperature. Protein size and purity were verified by 12% (*w*/*v*) SDS-PAGE and Coomassie Brilliant Blue staining. Protein concentration was assessed by measuring the optical density at 280 nm using an extinction coefficient of 14,565 M^−1^ cm^−1^, as estimated by the ProtParam tool [58].

The fractions containing Hsp60 were pooled and treated for 2 h on ice, with 20 mM of EDTA to induce the dissociation of Hsp60 oligomers [7,8]. Then, the Hsp60 fractions were transferred in 50 mM of Tris-HCl buffer, pH 7.7, 0.3 M NaCl, and concentrated by using the 10 kDa Amicon^®^ Ultra-Centrifugal Filters (Millipore, Sigma Aldrich Merck Darmstadt, Germany). After that, aliquots of the protein solution were loaded onto a Superdex 200 increase 10/300 GL (GE Healthcare) column equilibrated with the same buffer and eluted at a 0.75 mL min^−1^ flow rate. A 500 μL sample loop was used. The peak at the highest elution time (Figure S2A), corresponding to a population of monomers and dimers, was collected, and stored in 50 mM of Tris-HCl pH 7.7, 0.3 M NaCl, 10% (*w*/*v*) glycerol, at −80 °C.

The column was calibrated using a protein standard mix of thyroglobulin, bovine γ-globulin, chicken ovalbumin, equine myoglobin, and vitamin B12, MW 670,000–1350 (Biorad, Hercules, CA, USA).

### 4.3. Human Hsp60 Oligomerization

The in vitro assembly reaction was performed according to the procedure described by Viitanen et al. [8], with some modifications. Briefly, the fraction of Hsp60 corresponding to monomers and dimers was thawed, and the concentration of glycerol was reduced to less than 1% by repeated dilutions with glycerol-free buffer and re-concentration by centrifugation at 7500× *g* with 10 kDa Amicon^®^ Ultra-Centrifugal Filter Units (Millipore). A portion of the final sample was taken and used for measurements of the protein in monomeric conformation. The chromatographic profile of this sample (Superdex 200 increase 10/300 GL (GE Healthcare) column, 50 μL loop, 0.75 mL min^−1^ flow rate) confirmed that the protein population was still in equilibrium between dimers and monomers (Figure S2B).

To start the oligomerization procedure, the buffer of the remaining part of the sample was replaced by 50 mM of Tris HCl, pH 7.7, 0.3 M NaCl, 20 mM KCl, 25 mM MgCl_2_, by adding the appropriate quantity of the same buffer with higher KCl and MgCl_2_ concentrations. The sample was incubated for 90 min at 30 °C in the presence of 4 mM of ATP (Sigma-Aldrich), and then injected into a Superdex 200 increase 10/300 GL (GE Healthcare) column to analyze the oligomer population (Figure S2C). Aliquots with the highest percentage of tetradecamers were collected and concentrated for use in subsequent experiments. The entire procedure was applied to an aliquot of a protein sample incubated without ATP, in which case no tetradecamers were observed.

### 4.4. Preparation of Aβ_40_ Samples

The synthetic peptide Aβ_40_ (AnaSpec, Fremont, CA, USA) was pretreated following the procedure of Fezoui et al. [59]. Stock aliquots were stored at −80 °C until use. Aβ_40_ samples were prepared in a cold room by dissolving the lyophilized peptide in 50 mM of Tris-HCl, 3% glycerol, 0.3 M NaCl, pH 7.7, at a concentration of about 70 μM. The sample was then filtered through 0.20 μm (Millex-LG, Millipore, Darmstadt, Germany) and 0.02 μm (Whatman, Maidston, UK) filters, to eliminate large aggregates. The peptide concentration was measured by tyrosine absorption at 276 nm using an extinction coefficient of 1.39 cm^−1^ M^−1^. The sample was then diluted at the working concentration of 50 μM by adding the appropriate amounts of buffer, and concentrated solutions of ThT and Hsp60 when required.

### 4.5. Atomic Force Microscopy (AFM)

Aliquots of Aβ_40_ samples with or without Hsp60, taken at the end of fibrillization kinetics, were deposited onto freshly cleaved mica surfaces (Agar Scientific, Assing, Italy) and incubated for up to 60 min at room temperature. Then, samples were rinsed with deionized water and dried under a low-pressure nitrogen flow. AFM measurements were performed using a Nanowizard III (JPK Instruments, Berlin, Germany) system mounted on an Eclipse Ti (Nikon, Japan) inverted optical microscope. Tapping mode AFM images were acquired in the air using a multimode scanning probe microscope driven by a nano-scope V controller (Digital Instruments, Bruker, Kennewick, WA, USA). Single-beam uncoated silicon cantilevers (type SPM Probe Mikromasch) were used. The drive frequency was between 260 and 325 kHz, and the scan rate was 0.25–0.7 Hz.

### 4.6. Differential Scanning Calorimetry (DSC)

DSC experiments were performed with a Nano-DSC instrument (TA Instruments, New Castle, DE, USA) equipped with 0.3 mL of capillary platinum cells. The unfolding of monomeric or oligomeric 15 μM Hsp60 was obtained within the temperature range 25–80 °C, at a 30 °C/h scan rate. Both the protein solution and buffer were degassed before loading in the respective cells. The instrumental baseline was obtained by a preliminary temperature scan, with both cells filled with degassed buffer. The so-called chemical baseline, which is the contribution due to the difference in the heat capacities of the native and unfolded states of the protein, was calculated by linearly extrapolating the pre- and post-unfolding baselines into the transition region and merging them in proportion to the unfolding progress [31]. This was subtracted to obtain the excess heat capacity.

The calorimetric enthalpy change of the unfolding transitions (Δ*H_cal_*) was obtained by integrating $C_{P}^{EX}$ over the temperature interval comprised between *T*_1_ and *T*_2_ values:

(1) $$\Delta H_{cal}=\int_{T_{1}}^{T_{2}}C_{P}^{EX}dT$$

*T_m_* was defined as the temperature value at which excess unfolding heat capacity is maximal. The van’t Hoff enthalpy was calculated by the relationship [60]:

(2) $$\Delta H_{vH}=4\;R\;T_{m}^{2}\;\frac{\Delta C_{P,\;max}^{EX}}{\Delta H_{cal}}$$

To deal with DSC profiles biased by the likely occurrence of aggregation upon folding, the simplest form of the Lumry–Eyring model [32] was applied in data analysis. In this model, the reversible transition from the native to the unfolded state is coupled to a conversion of the unfolded molecule into an altered “final” state, from which it cannot fold back. This conversion is a kinetic process governed by an activation energy, *E_A_*. If the transition to the final state is fast enough, the population of unfolded molecules is very low, and the transition from the native to the final state can be modeled as:

$$N\overset{k}{\to }F$$

The temperature dependence of the rate constant, *k*, is given by the Arrhenius equation:

(3) $$k=exp\left[-\frac{E_{A}}{R}\left(\frac{1}{T}-\frac{1}{T_{A}}\right)\right]$$

where *T_A_* is the temperature at which *k* = 1. By applying the kinetic analysis developed by Sanchez-Ruiz and co-authors [32,61], C_P_ trace was fit to the expression:

(4) $$C_{P}=C_{P}^{Pre}+\left(C_{P}^{Post}-C_{P}^{Pre}\right)\left(1-X_{N}\right)-\Delta H\;\left(dX_{N}/dt\right)$$

where *X_N_* is the fraction of native molecules, ΔH is the unfolding enthalpy, and $C_{P}^{Pre}$ and $C_{P}^{Post}$ are the temperature-dependent pre- and post-transition baselines. *X_N_* and ((*dX_N_*)/*dt*) are expressed as:

(5) $$X_{N}=\exp(-exp\left(\frac{E_{A}\;\left(T-T_{m}\right)}{RT_{m}^{2}}\right)$$

(6) $$dX_{N}/dT=-\frac{E_{A}}{RT_{m}^{2}}\;exp\left(\frac{E_{A}\;\left(T-T_{m}\right)}{RT_{m}^{2}}\right)\exp(-exp\left(\frac{E_{A}\;\left(T-T_{m}\right)}{RT_{m}^{2}}\right)$$

A downward temperature scan followed this to check the reversibility of the unfolding transition.

### 4.7. Circular Dichroism Spectroscopy (CD)

Circular dichroism (CD) spectra of 15 μM of Hsp60 in either monomeric or multimeric form were recorded at increasing temperatures using a CD spectrometer (JASCO J-810, Jasco Europe, Cremella, LC, Italy)), equipped with a Peltier unit for temperature control. The cell path length was 0.2 mm. The CD signal at a 225 nm wavelength (typical choice for following the thermal denaturation of the alpha-helix) was continuously monitored during the temperature scan, from 20 to 90 °C at a 30 °C/h rate. Spectra in the 190–260 nm wavelength interval were collected every 5 °C (Figure S1). Each CD spectrum was the average of 10 scans subtracted by the solvent contribution. Data are presented in molar absorbance units per residue.

The CDPro software package [35] was used to evaluate the secondary structure elements and their changes as a function of temperature. The relative contribution of alpha-helix, beta-sheet, turns, and disordered elements was determined by averaging the results from CONTIN, CDSSTR, and SELCOM 3 programs.

### 4.8. Thioflavin T Fluorescence Assay for Aβ_40_ Fibrillization

The change in ThT fluorescence emission during Aβ^40^ aggregation kinetics was monitored using a JASCO FP-6500 spectrometer. The excitation and emission wavelengths were 450 and 485 nm, respectively. The concentration of ThT was 12 μM. The concentration of Hsp60, if present, was 2 μM. The sample was placed in the thermostated cell compartment at 37 °C and continuously sheared at 200 rpm using a magnetic stirrer (mod. 300, Rank Brothers Ltd., Cambridge, UK).

### 4.9. Dynamic and Static Light Scattering

Samples of monomeric or multimeric Hsp60 at a 15 μM concentration were directly filtered into a dust-free quartz cell and placed in the thermostatic cell compartment of a Brookhaven Instruments BI200-SM goniometer. The temperature was controlled within 0.1 °C using a thermostatic recirculating bath. Samples were allowed to equilibrate at 20 °C before beginning a temperature scan from 20 to 80 °C at a scan rate of 8 °C/h. The scattered light intensity and time autocorrelation function were measured at a scattering angle of 90° using a Brookhaven BI-9000 correlator and a 50 mW He–Ne laser tuned to a 632.8 nm wavelength. In dynamic light scattering (DLS) experiments, the correlator was operated in the multi-τ mode, and the experimental duration was set to 3 min. Static light scattering data were corrected for the background scattering of the solvent and normalized by using toluene as the calibration liquid. The autocorrelation function was analyzed using the cumulant method [62] to derive a z-averaged translational diffusion coefficient, which was converted to an average apparent hydrodynamic radius of an equivalent sphere, R_H_, through the Stoke–Einstein relationship: D_z_ = kBT/6πηR_H_.

## Figures and Tables

**Table 1:** Fit parameters of Hsp60 thermal unfolding to the two-state irreversible model.

	ΔH (kJ mol^−1^)	T_m_ (°C)	E_A_ (kJ mol^−1^)
Monomeric form	276	42.4	298
Tetradecameric form	188.4	59	381

## Data Availability

Data are contained within the article or Supplementary Materials.

## Supplementary Materials

The supporting information can be downloaded at: https://www.mdpi.com/1422-0067/24/9/7847/s1.
