# Supplementary material for: Oligomeric State and Holding Activity of Hsp60
Source: Int J Mol Sci. 2023 Apr 25;24(9):7847. doi: 10.3390/ijms24097847 (PMC10177986; doi:10.3390/ijms24097847)

Oligomeric state and holding activity of Hsp60

Celeste Caruso Bavisotto, Alessia Provenzano, Rosa Passantino, Antonella Marino Gammazza, Francesco Cappello, Pier Luigi San Biagio, Donatella Bulone

Supplementary Materials

Figure S1. CD spectra at varying temperature for 15 μM Hsp60 in monomeric (left panel) and tetradecameric (right panel) form.

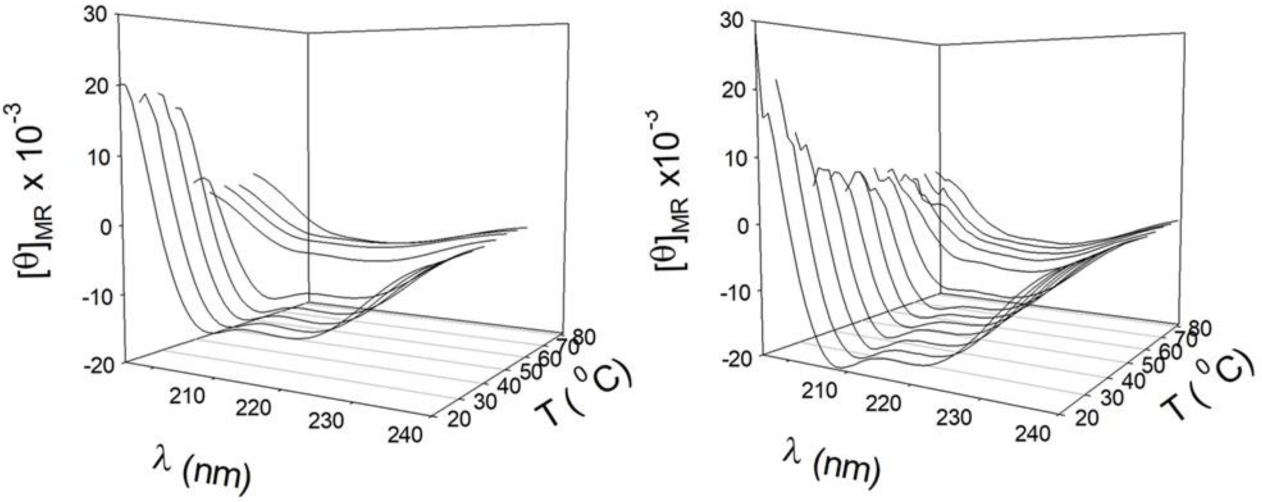

Figure S2. Panel A: Size exclusion chromatographic profile of recombinant Hsp60 expressed in bacteria and purified as described in the text. The peak with the smallest elution volume is influenced by the presence of species larger than the column pore size (200 nm) and is therefore ignored. The peak with the largest elution volume, corresponding to protein in dimeric and monomeric form, was collected and stored at -80 ^{0}C. A small fraction of heptamers is detectable at 10 ml elution volume. Panel B: Chromatographic profile of the mono-dimeric fraction after thawing and resuspension in solution. Panel C: Chromatographic profile of the mono-dimeric fraction after treatment with ATP. The presence of larger species with dimensions corresponding to those of tetradecamers is now evident.

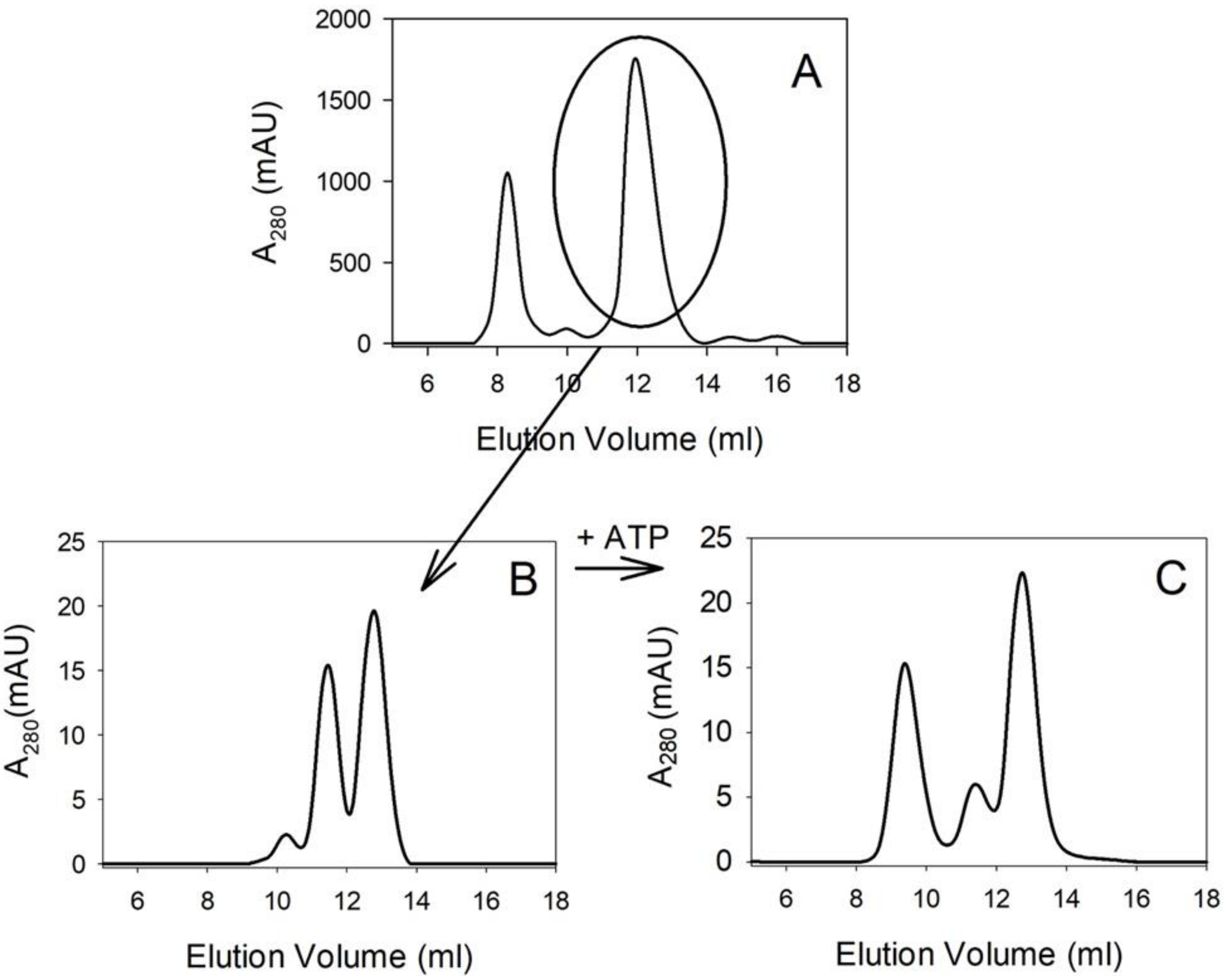

Supplement: Supplementary file 1 [file ijms-24-07847-s001.zip › ijms-2343633-supplementary.pdf]
